# *In vitro* evaluation of the neuroprotective potential of *Olea dioica* against Aβ peptide-induced toxicity in human neuroblastoma SH-SY5Y cells

**DOI:** 10.3389/fphar.2023.1139606

**Published:** 2023-05-10

**Authors:** Pratap G. Kenchappa, Yalpi Karthik, Poornima D. Vijendra, Raghavendra L. S. Hallur, Ajay S. Khandagale, Ashok K. Pandurangan, Sathisha G. Jayanna, Mohammed Ali Alshehri, Abdulrahman Alasmari, Samy Sayed, Manjula Shantaram, Muntazir Mushtaq

**Affiliations:** ^1^ Department of Studies and Research in Biochemistry, Jnana Kaveri Post Graduate Centre, Mangalore University, Kodagu, Karnataka, India; ^2^ Department of Studies and Research in Microbiology, Jnana Kaveri Campus, Mangalore University, Kodagu, Karnataka, India; ^3^ Department of Studies in Food Technology, Davangere University, Davangere, Karnataka, India; ^4^ Center for Biotechnology, Pravara Institute of Medical Sciences (Deemed to Be University), Ahmednagar, Maharashtra, India; ^5^ SDM Research Institute for Biomedical Sciences (SDMRIBS) Shree Dharmasthala Manjunatheshwara University, Dharwad, Karnataka, India; ^6^ School of Life Sciences, B.S. Abdur Rahman Crescent Institute of Science and Technology, Chennai, India; ^7^ Department of Biochemistry, Jnanasahyadri, Kuvempu University, Shivamogga, Karnataka, India; ^8^ Department of Biology, Faculty of Science, University of Tabuk, Tabuk, Saudi Arabia; ^9^ Genome and Biotechnology Unit, Faculty of Sciences, University of Tabuk, Tabuk, Saudi Arabia; ^10^ Department of Science and Technology, University College-Ranyah, Taif University, Taif, Saudi Arabia; ^11^ MS Swaminathan School of Agriculture, Shoolini University of Biotechnology and Management Sciences, Solan, Himachal Pradesh, India

**Keywords:** alzeheimer’s disease, olea dioica roxb, amyloid, beta, SHSY-5Y, GC-LCMS, acetylcholinesterase

## Abstract

Alzheimer’s disease (AD) is a type of neurodegenerative disease, associated with the hastening of ROS, acetylcholinesterase (AChE) activity, and amyloid β peptides plaques in the brain. The limitations and side effects of existing synthetic drugs incline toward natural sources. In the present communication active principles of methanolic extract of *Olea dioica* Roxb, leaves are explored as an antioxidant, AChE inhibitor, and anti-amyloidogenic. Furthermore, neuroprotection against the amyloid beta-peptide has been studied. The bioactive principles were identified by GC-MS and LC-MS and further subjected to antioxidant (DPPH and FRAP) and neuroprotection (AChE inhibition, ThT binding, and MTT assay, DCFH-DA and lipid peroxidation (LPO) assay using neuroblastoma (SHSY-5Y) cell lines) assays. Methanolic extract of *O. dioica* Roxb, leaves was found to contain polyphenols and flavonoids. *In vitro* assays exhibited potential antioxidant and anti-AChE (˃50%) activities. ThT binding assay indicated protection against amyloid-beta aggregation. MTT assay, Aβ1-40 (10 µM) with extract increase the cell viability (˃50%) and showed significant cytotoxicity to SHSY-5Y cells. ROS level (˃25%) significantly decreased in the Aβ1-40 (10 µM) + extract (15 and 20 μM/mL) and LPO assay (˃50%) suggesting prevention of cell damage. Results advocate that *O. dioica* leaves are a good source of antioxidants, anti-AChE, and anti-amyloidogenic compounds which may be further evaluated as a natural medicine for the treatment of AD.

## Introduction

Among neurodegenerative diseases, common dementia or Alzheimer’s disease (AD) attributes to the loss of neurons in different parts of the brain. The build-ups of amyloid β peptide plaques are commonly seen in the brains of people with AD. Moreover enhanced oxidative stress in neural cells causes cell damage and cell death that results in progressive AD with mild to the severe cognitive deficit (Querfurth et al., 2010; Thompson et al., 2012). Low levels of acetylcholine (ACh), a neurotransmitter of the parasympathetic nervous system are another attribute of AD.

Acetylcholinesterase (AChE), is a serine hydrolase that performs ACh hydrolysis to acetate and choline resulting in the termination of impulse transmission at cholinergic synapses in the brain of AD patients (R. Wang & Xi, 2005). AChE stimulates the development of amyloid fibrils and produces AChE-Aβ complexes (Hardy & Selkoe, 2002). These AChE-Aβ complexes accelerate neurotoxicity leading to greater neuro-degeneration. Inhibition of AChE activity has been acknowledged to be a significant therapeutic approach to controlling AD progression. Synthetic anti-cholinesterases or AChE inhibitors such as galantamine, donepezil, memantine, rivastigmine, tacrine, and physostigmine are currently known drugs for the management of AD (Js et al., 2015). Although these anti-cholinesterases exert toxic effects while pharmacological manipulation of the AChE activity in AD patients. Toxic effects include vomiting, nausea, diarrhea, headache, anorexia, and abdominal pain.

Plants are a valuable resource in human medicine, especially for the manufacture of pharmaceutical and herbal drugs. These plant-based drugs are increasingly appreciated to treat many human ailments nowadays (Grossi et al., 2013). The identification of new pharmaceutical leads and the safe use of plant-based drugs are both aided by phytochemical and pharmacological characterizations. The increase in the rate of allopathic medicines, the reliable cure for many chronic diseases, the easy availability of drugs from natural sources, and the prohibitive cost of neuroprotective allopathic drugs with added side effects are some of the reasons for the popularity of plant-based medicines. Moreover, products made from plants are less expensive and have fewer adverse effects (Bhandary et al., 1995; Harsha et al., 2003) In this perspective, many phytochemicals from medicinal plants are investigated for anti-cholinesterases or anti-AChE activity and the most popular field of research in the management of AD as the prevalence of AD has increased (Adewusi & Steenkamp, 2011; Chapter 5 Purification and Characterisation, n.d.; Luccarini et al., 2015). Ethno-medicinal plants such as *Bacopa monera*, *Mentha longifolia* L.), *Ocimum basilicum* L., and others are used to treat neurological illnesses including AD recently (Pratap et al., 2017; Pratap & Shantaram, 2020a).

*Olea dioica* Roxb or Rose Sandal Wood is a medicinal angiosperm tree commonly found in the Western Ghats of the Indian subcontinent. It is an important ethnomedicinal tree and belongs to the family of Oleaceae. Earlier studies reported antibacterial, antioxidant, aphrodisiac, and cytotoxic properties from *Olea dioica* Roxb, (Rao & Naika, 2015). According to Siddha medicine, the roots of *Olea dioica* are effective in the treatment of cancer and snake bite (Ashwathanarayana & Naika, 2017). The fruits of *Olea dioica* are used to cure skin diseases (Yesodharan & Sujana, 2007). The plant extract of *Olea dioica* exhibits neuroprotective effects in *Drosophila* against acrylamide-induced neurotoxicity (Pratap et al., 2021). According to a literature survey, *Olea dioica* Roxb, is a very important ethnomedicinal plant owing several pharmacological activities and could be a source of anti-AChE and anti-amyloidogenic compounds which could be a promising candidate for creating new drug lead for the controlling progression of AD in humans. With this impetus, the current study was planned to isolate, identify and characterize different phytochemicals for anti-oxidative, anti-AChE, and anti-amyloidogenic properties.

## Materials and methods

### Chemicals

Dulbecco’s modified eagle’s medium and FBS were purchased from Gibco (United States), and SHSY5Y cells were from ATCC. 5,5′-dithiobis-(2-nitrobenzoic acid) (DTNB), 2′,7 di-chloro fluorescein di-acetate (DCFD), SDS, TBA, TLC plates, Aβ1-40 peptide, acetylcholine iodide (ACTI) and MTT were from (Merck-Sigma Aldrich) and other chemicals were purchased from Hi-media.

### Plant extract preparation

*O. dioica* Roxb plant leaves were collected from Pilikula, Mangalore, Karnataka. After two rounds of distilled water rinsing, the leaves were left to dry for 23 days in the shade. The dried leaves were then pulverized into a fine powder using a mixer grinder. The powder (50 g) was Soxhlet-extracted with methanol for 12 hs, and it was kept at room temperature (RT) in brown bottles.

### Screening for phytochemicals

The plant leaves extractwas then subjected to a screening of different phytochemicals as flavonoids, saponins, tri-terpenoids, alkaloids, phenolic compounds, reducing sugars, steroids, proteins, carbohydrates, etc. Using the procedure reported previously (Pratap and Shantaram, 2020a).

### Spectral analysis

#### GC-MS analysis

Shimadzu 17A GC with Head Space Sampling System HSS-4A and an HP-5MS column (30 m 0.25 mm 0.1 m) were used to perform the analysis of the active fractions of the methanolic extract using gas chromatography-mass spectrometry (GCMS-S80) (Attimarad et al., 2011) (The sample was injected in split mode at 220°C, and the transfer line was set to 240°C. Helium was used as the carrier gas at a constant flow rate (1 mL/min). The temperature was ramped up from 60°C to 260°C at the rate of 3°C/min and then exposed at 260°C for 25 min in full scan mode, m/z (mass-to-charge ratio) 20–600. Electron impact ionisation was employed (70 eV), and the MS (mass spectrometry) ion source was maintained at 240°C. Compounds were identified by their gas chromatography retention times and mass spectra using standard compounds for comparison with methanolic fraction’s GC-MS graph (Pratap et al., 2020a; Pratap et al., 2020b).

#### LC-MS analysis

Instruments for LC-MS (Agilent 1100 LC, Bruker-make MS model Esquire 3000) having an ESI (electrospray ionisation), an APCI (atmospheric pressure chemical ionisation), and a PDA detection source with a mass range of 100 amu in quadrupole and 10,000 amu (Jain et al., 2016). The mass-charge ratio and molecular mass of the methanolic fractions were determined by using the spectrum database for organic compounds in MassBank, which was then utilised to identify the mass fragmentations (Pratap et al., 2020b; Gk et al., 2022).

#### FTIR analysis

By using FTIR analysis (Bruke), which scans spectra in the 4000–500 cm^1^ range, the functional groups on the methanolic fractions were identified. The sample was formed by uniformly dispersing the methanolic fractions within a matrix of KBr (Owokotomo et al., 2015). The intensity bands were compared to standard values in order to identify the functional groups (Pratap et al., 2020b; Gk et al., 2022).

#### Determination of total flavonoid content

The total flavonoids in the methanolic fractions were estimated using colorimetric techniques (Sugimoto et al., 2000; Adebiyi and Olayemi, 2017). A clean test tube with 2% AlCl_3_ solution (0.5 mL), 0.25 mL of distilled water, and 200 µL (1 mg/mL) of methanolic fraction was added. The absorbance at 510 nm was measured after 1 hour incubation period at room temperature. Using quercetin (1 mg/g), a positive control, the total flavonoid content was determined. The equation employed was y = 0.0255x, R2 = 0.9822, where x is the absorbance and y is the positive control of quercetin equivalent (mg/g), based on the calibration curve (Pratap et al., 2020a).

### Antioxidant assay

#### DPPH assay

DPPH assay for antiradical activity was performed by the Brand-Williams method (Cuvelier & Berset, 1995). In order to prepare fresh DPPH, 7.8 mg of DPPH were dissolved in 20 mL of methanol and stored at −20°C. One millilitre of 0.1 mM solution of DPPH +100 μL of the samples at different concentrations (20–100 μg/mL) was taken. The mixture was incubated in the dark for 15 min and the absorbance was read at 517 nm. Quercetin was used as standard and % inhibitions were calculated using the given formula.

$$Inhibition\;of\;DPPH\;radical\;\left(\%\right)=\frac{Ab-As}{Ab}\;X100$$

#### FRAP (ferric-reducing antioxidant power) assay

To assess antioxidant power of leaf extract of *O. dioica,* Benzie and Strain method (Benzie and Strain, 1996) with little modifications were used. The assay mixture was prepared by 150 mM of acetate buffer (pH 3.6), 20 mM ferric chloride hexa hydrate (FeCl_3_.6H_2_O) in the proportion of 10:1:1 and 10 mM of TPTZ in 40 mM hydrochloric acid at RT. Then 5 μL leaves extract (1mg/1 mL) was added to a freshly prepared working FRAP reagent (3.95 mL), mixed carefully, and incubated at RT for 30min. At low pH ferric ions were reduced to ferrous ion in the presence of leaves extract and blue coloured ferrous-tri-pyridyl tri-azine complex were formed. The absorbance values were measured at 593 nm against the blank.

#### AChE inhibition by bioautographic enzyme assay

According to the bioautographic approach (Devika & Koilpillai, 2014; Pratap et al., 2020a), the acetylcholinesterase inhibitory activity of the methanolic fractions was discovered using TLC. The methanolic fraction was spotted (10 g/mL) and developed with a standardising mobile-phase (CHCl3:MeOH, 9.5:0.5) before being dried at room temperature on a silica gel-coated TLC plate, 2.5 mm F-254, 10 10 cm (Merck, Germany). The plates were then sprayed with freshly made AChE (Electric eel AChE enzyme; Sigma-Aldrich) enzyme solution, and they were let to sit at room temperature for 3 min. They were then sprayed till they reached saturation level with 3 mM DTNB or Ellman’s reagent and 15 mM acetylcholine iodide (ATCI) solution in phosphate buffer (pH 7.2). In order to establish the presence of putative AChE inhibition zones, the plates were later dried at room temperature for 1 min, resulting in colourless or white specks on a yellow background. After measuring the Rf value of the plant bioactive components that were calculated for the separation of the molecule in preparative scale, the spots on the plates were confirmed for being possible AChE inhibitors and recognised as clear zones against a yellow background (Marston et al., 2002; Pratap et al., 2020a; Pratap et al., 2020b).

#### AChE inhibition assay by Ellman’s method

The Ellman spectrophotometric approach (Ellman et al., 1961) (Wal et al., 2011) was used to measure the AChE inhibition activity, albeit with a little modification. Briefly, 20 μL of AChE (3 U/mL) solution, 3 mL of 0.1 M Tris-HCl buffer (pH 8.0), 100 μL of methanolic fractions at various concentrations (20, 40, 60, 80, 100, and 120 g/mL), and incubated at RT for 15 min and later 50 μL of 3 mM DTNB was added. When a yellow colour develops, 50 μL of 15 mM AChI (acetylthiocholine iodide) was then added to start the reaction. The positive control (1 mg/mL) utilised was galantamine. With a UV-visible spectrophotometer, the mixture’s absorbance was determined at 412 nm (Beckman Coulter, United States). This assay was performed in triplicate. By comparing the enzyme activity to the negative control, the percentage inhibition was computed using the following formula:

$$Inhibition\%=\frac{A_{0}-A_{1}}{A_{0}}X100$$

#### ThT assay of disaggregation of preformed Aβ_1-40_ aggregates

Thioflavin T (ThT) fluorescence assay is frequently used to evaluate the fibrillogenesis (amyloids) rate. A ThT molecule produces fluorescence upon binding to amyloids and could be used to monitor *in vitro* amyloid fibril aggregation (Xue et al., 2017). The leaves extracts (10–100 μg/mL) were incubated with 10 µM of Aβ_1-40_ (40 µL) at 37°C for 1 day. Then, 100 µL of ThT (10 µM in PBS buffer, pH 7.4) was added. An FP-6200 spectrofluorometer (Shimadzu RF-5000) was used to assess the amounts of ThT fluorescence in the samples and the fluorescence was measured after 30 min at the wavelengths of excitation and emission Ex_450nm_ and Ex_483nm_ respectively along with the positive control (Galantamine + Aβ).

### Cell culture

The neuroblastoma (SHSY-5Y) cells were cultured in a DMEM (Dulbecco’s Modified Eagle Medium) medium and supplemented with 10% FBS, antibiotics, and growth factor, at 37°C in a humid atmosphere with 5% CO_2_.

#### Preparation of amyloid-beta (Aβ _1–40_)

Aβ _1–40_ stock solution (0.1 mg/mL) was prepared in milli-Q. Before using, Aβ _1–40_ solution was incubated at 37°C in a water bath for about 7–8 h with mixing (every 1 h) for aggregation and then diluted in the medium. After aggregation, the solution was divided into aliquots and kept at −20°C in sterile Eppendorf tubes.

### MTT assay

SHSY-5Y cells (3 × 10^4^cells/well) were seeded in 96 well plates. The medium was replaced every 24 h so that the cells become adherent. Further, the cells were added to a serum-free medium and treated with or without leaves extract + Aβ_1-40_ at different concentrations and then incubated for 24 h after which the cell viability was checked and measured by MTT (5 mg/mL) assay. The MTT assay (Dalberto et al., 2020) was performed by adding MTT solution to each well and incubating for 4 h at 37°C. After removing the MTT solution, 100 μL of DMSO (100%) was added to each well, and absorption was read at 570 nm using a microplate reader. Cell viability data were compared in two ways: treated cells *versus* untreated cells with Aβleaves extract, and leaves extract + Aβ. The concentration was selected based on the MTT results.

$$\%\;of\;cell\;viability=\frac{OD\;of\;test}{2OD\;of\;control}\;X100$$

#### Dichloro-dihydro-fluorescein diacetate (DCFH-DA) assay

Reactive Oxygen Species (ROS) levels were monitored using DCFH-DA assay (Aranda et al., 2013). SHSY-5Y cells (25 × 10^4^) were seeded into 96 well plates and allowed to grow for 24 h. The cells were then washed with PBS, replaced with fresh medium (no FBS), and incubated for 24 h with 10 μM Aβ_1-40_ solution in the presence or absence of leaf extract at different concentrations. The cells were further incubated with DCFH-DA (5 μM) at 37°C for 30 min and washed with cold phosphate-buffered saline (PBS 7.4 pH). The fluorescence intensity was measured at wavelengths of 490 nm for excitation and 520 nm for emission in the microplate reader (Perkin Elmer LS 55 Luminescence Spectrometer). After completion of the process, the results were expressed as Relative Fluorescence Units (RFU).

#### Lipid peroxidation assay

Lipid peroxidation assay was performed using neuroblastoma cells (SHSY-5Y) by a method described earlier with slight modification (Sereia et al., 2019). The SH-SY5Y cells were seeded with the same density and same treatment as that of the ROS assay described above. The lipid peroxidation was evaluated to the extract during the exposure of SHSY-5Y cells at 20 μg/mL for 2 h. After that, it was combined with 10 μM of Aβ_1-40_ and incubated for another 24 h using the diphenyl-1-pyrenylphosphine (DPPP, 45 µM) probe for 15 min at 35°C and then the fluorescence was measured at λex_355_ nm and λem_460_ nm.

#### AChE activity

Ellman assay was used to measure the AChE activity in a 96-well microplate (Ellman et al., 1961). The SHSY-5Y cells were seeded at the same density and same treatment (ROS assay described above). After treatment, the cell culture media was collected and the cells were scraped in a cold phosphatebuffer. The reaction mixture containing 60 µL of cell scraped, 30 µL of DTNB (0.5 mM), and 30 µL of AChI (1 mM) was incubated for 5 min. Absorbance was measured at 412 nm for up to 10 min. The AChE activity was expressed as Unit/mg protein.

#### Statistical analysis

The differential significance of the results obtained was determined by one-way ANOVA followed by Bonferroni’s multiple comparisons test at the 0.05 level. All values are presented with Means ± SD, except where otherwise indicated. Statistical analysis was carried out using GraphPad Prism 8. Comparisons between two groups were performed using a one-way analysis of variance, whereas the comparisons among multiple groups were performed with one-way ANOVA. *p* < 0.05 was considered to indicate a statistically significant difference.

## Results

Plants produce a different array of chemical compounds among them certain bioactive compounds like phenolics, anthocyanins, and flavonoids, etc. Have gained extensive approval for their physiological functions, which include free radical scavenging, anti-diabetic, and anti-cancer effects (Bhandary et al., 1995). The present study has revealed the bioactive constituents and neuroprotective potential of the leaf extract of *Olea dioica.* Earlier we have demonstrated *in vitro* anti-oxidant and anti-AChE potential of column-purified fractions of methanol extract of leaves (Pratap et al., 2020a). Here we specifically focusedon the detailed phytochemical analysis and characterization of the methanol extract of *O. dioica* Roxb leaves. We have also explored *in vitro* anti-AChE, anti-oxidant, anti-amyloids, and neuroprotective potential of *O. dioica* Roxb leaves extract using neuroblastoma (SHSY-5Y) cells.

### Phytochemical analysis

During the primary phytochemical screening, methanolic extract of *O. dioica* Roxb leaves was found to contain saponins, tri-terpenoids, coumarins, flavonoids, and phenolic compounds. However, proteins, sugars, and steroids were absent (data is not represented). A literature survey illustrates positive correlations between antioxidant potential and phenolics and flavonoids contents (Yang and Gadi, 2008; Okpuzor et al., 2009). These natural compounds are found to prevent the development of AD pathology *in vitro* and *in vivo* experimental settings (Hamaguchi et al., 2009). Here, the total phenolic and flavonoid content of the methanolic leaf extract of *O. dioica* Roxb was determined from the calibration curve of standard Gallic acid and Quercetin which was then expressed as mg equivalent of Gallic acid and Quercetin per gram of dry extract respectively. The phenolic content was found to be 26 μg/mL (SD ± required) gallic acid equivalents/g, whereas the flavonoids were found to be 18.3 μg/mL (Mean ± SD) quercetin equivalent/g. Besides, phenolics and flavonoids the leaves extract were found to contain tannins, and saponins (data is not shown).

### GC-MS and LC-MS analysis

**FIGURE 1:**
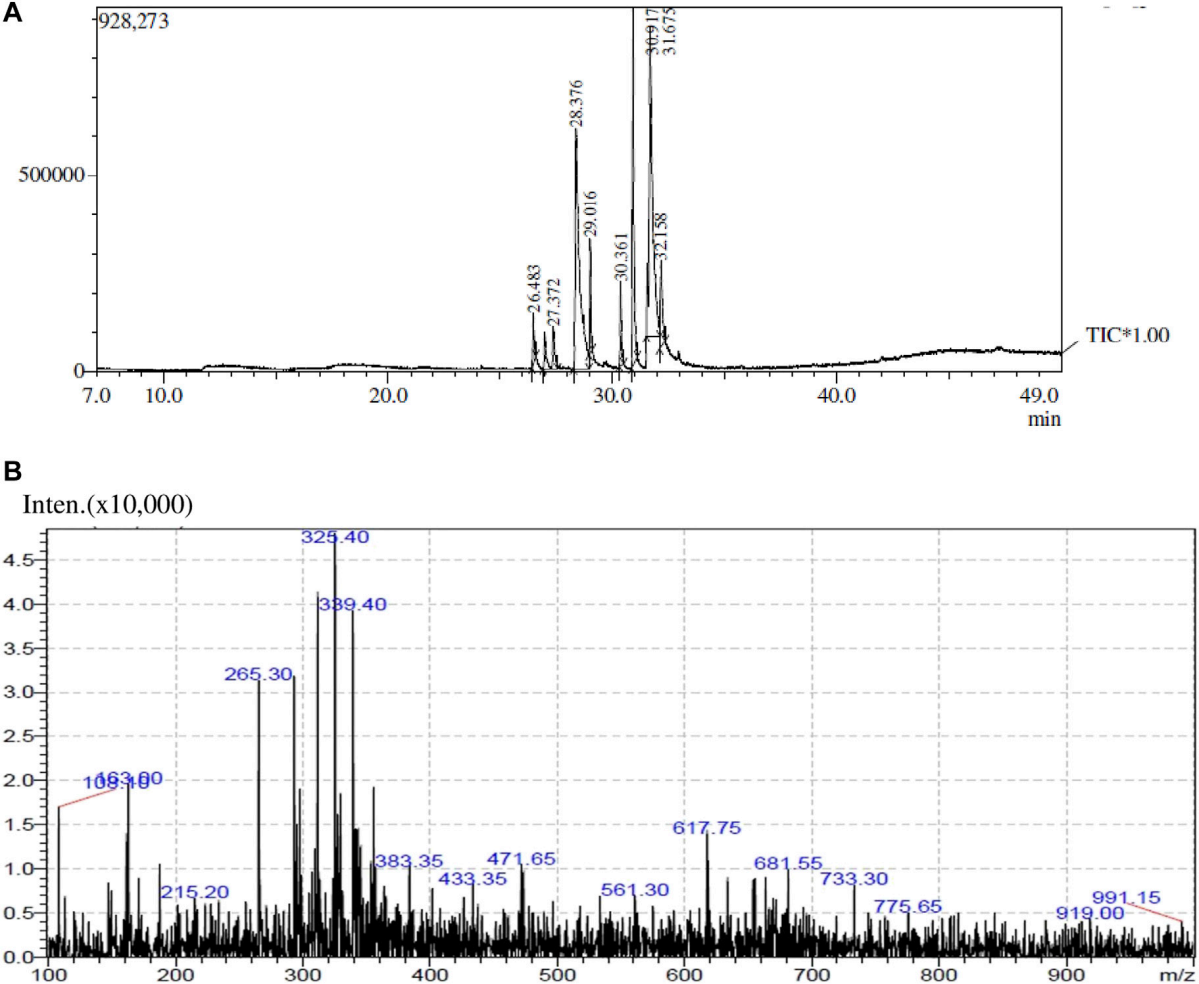
GC-MS **(A)** and LC-MS **(B)** analyses of methanol extract of *O. dioica* leaves.

The GC-MS analysis spectra of the extract of *O. dioica*Roxb leaves is shown in Figure 1 exhibited multiple peaks that represented the presence of distinct phytochemical components. A comparison of the MS constituents with the NIST library confirmed the identity of eight phytochemicals. The LC-MS spectra also showed different peaks and the masses compared with the mass bank library confirmed the identity of four compounds (Tables 1, 2).

**TABLE 1:** Identified components present in GC-MS and biological activity of the components.

**S. No.**	**Compound Name**	**Formula**	**Molecular Weight**	**Component RT**	**Biological Activity**	**Structure**
1.	Hexadecanal	C_16_H_32_O	240.2	16.5304	Not reported	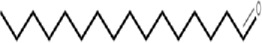
2.	Octadecanal	C_18_H_36_O	268.4	18.5544	Antibacterial activity, anti-cancer activity (Mohammed et al., 2016)	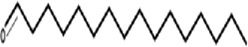
3.	6-Octadecenoic acid, (Z)-	C_18_H_34_O_2_	283.5	19.6524	Cancer preventive, Insectifuge (Anand Gideon, 2015)	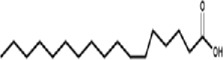
4.	Octadecenoic acid	C_18_H_36_O_2_	282.4	19.8364	Antimicrobial Activity (Rahman et al., 2014)	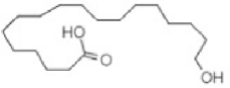
5.	Lupeoltri fluoroacetate	C_32_H4_9_F_3_O_2_	426.7	21.7475	trifluoroacetate, anti-tumour, anti-inflammatory (Arora et al., 2017)	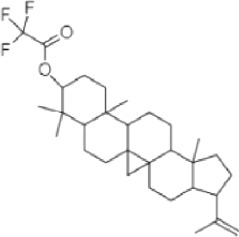
6.	12-Oleanen-3-yl acetate (3Alpha)	C_32_H_52_O_2_	468.7	22.0146	Not reported	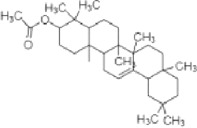
7.	Urs-12-en-3-ol, acetate-	C_32_H_52_O_2_	468.8	22.1155	Antimicrobial activities (Lazreg-Aref et al., 2012)	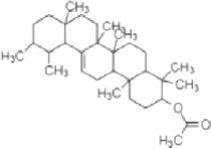
8.	Lu-20(29)-en-3-ol, acetate, (3.beta.)-	C_32_H_52_O_2_	436.7	23.9495	Anti-inflammatory (Wal et al., 2011)	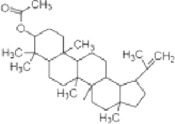

**TABLE 2:** The identified components present in GC-MS and LC-MS analysis and biological activity of the components and the mass compared with the MassBank database.

**S. No.**	**Compound name**	**Molecular formula**	**Molecular Weight**	**Biological activity**	**Structure**
1.	Isocryptotanshinone	C_19_H_20_O_3_	296	Antibacterial, anti-oxidative and anticancer activities (Guo et al., 2016)	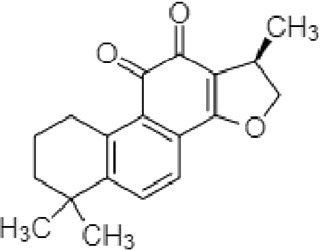
2.	Ginkgolic acid	C_22_H_34_O_3_	346	Antimicrobial activities, antioxidant and cytotoxic activities (Hua et al., 2017)	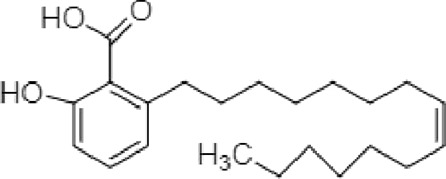
3.	Soya cerebroside	C_40_H_75_NO_9_	713	Antibacterial and antioxidant activities, anti-cancer activity (Liu et al., 2017)	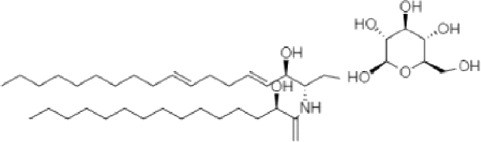
4.	Loganetin	C_11_H_16_O_5_	228	Antimicrobial and antifungal activity (Moreira et al., 2015)	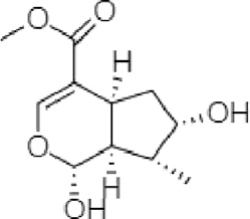

### FTIR analysis

**FIGURE 2:**
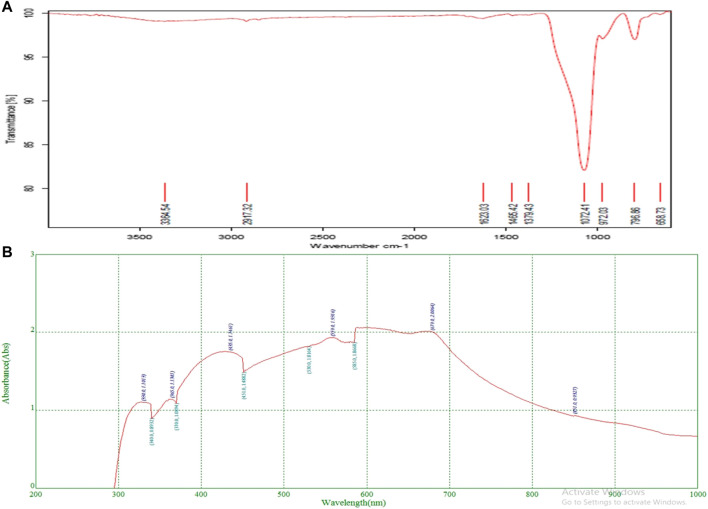
FTIR analysis **(A)** and UV- Absorbance **(B)** of methanol extract of *O. dioica* leaves.

The functional groups and chemical components of structural components can be identified using the high-resolution analytical technique known as FTIR (Hussain et al., 2009). The functional groups of bioactive components in the leaf extract were essentially discovered by the FTIR measurement (Figure 2). FTIR results showed the band in the range of 3500–500 cm^−1^. The stretch and vibration of each band show the existence of functional groups such as O-H, which indicate alcohols and phenols. The FTIR analysis indicated the existence of the functional groups listed in Table 3. Specifically functional groups werealcohols, carboxylic acids, alkenes, amines, phenols, aldehydes, quinines, amides, anhydrides, and organic halogen compounds. All these compounds belong to the plant secondary metabolites.

**TABLE 3:** FTIR peak values and functional groups of plant leaf extract.

Extracts	Peak value	Functional group	Functional group name	Vibrations
Extract of *Olea dioica* Roxb	784.63	C-I	Haloalkane	Stretch
	903.22	-	-	-
	1092.49–1220.74	C-F	Haloalkane	Bending
	1359.22–1421.56	C-O, C-F	Alcohols, ethers,esters, and haloalkane	Bend out- of- plane
	1708.79	C=O	Acid, saturated	**-**
	3004.45	C-H	Aromatic	Bending
	3532.66	O-H	Alcohols and Phenols	Bending

### UV-VIS analysis

The phytoconstituents present in methanolic leaves extract of *Olea dioica* Roxb., were identified by the UV-VIS spectra analysis, and were performed at the appropriate baseline, 200–1000 nm wavelength. (Figure 2). The flavonoids and their derivatives are represented by absorption peaks at 200–400 nm (Attimarad et al., 2011) The flavonoids have only two absorption maxima in the range of 230–285 nm and 300–350 nm. However, alkaloids, flavanols, flavonoids, and phenolic acid are known to have a presence range of 270–670 nm (Table 4). The crude extract included alkaloids, flavanols, flavonoids, and phenolic acid. Information on the secondary metabolites of the plants is provided by these intensity measurements (Domenici et al., 2014).

**TABLE 4:** Absorption peaks ranging from 200 to 300 nm and 400–700 nm indicating the presence of plant compounds.

	Absorbance range	Type of compound present
Plant crude extract	200–300 nm	Represents the presence of flavonoids and their derivatives
	400–700 nm	Represents the presence of Phenolic acid, flavonoids, flavanol and alkaloids

The phytoconstituents present in methanolic leaves extract of *Olea dioica* Roxb., were identified by the UV-VIS spectra analysis performed at the appropriate baseline, 200–1000 nm wavelength.

### Antioxidant activity

**FIGURE 3:**
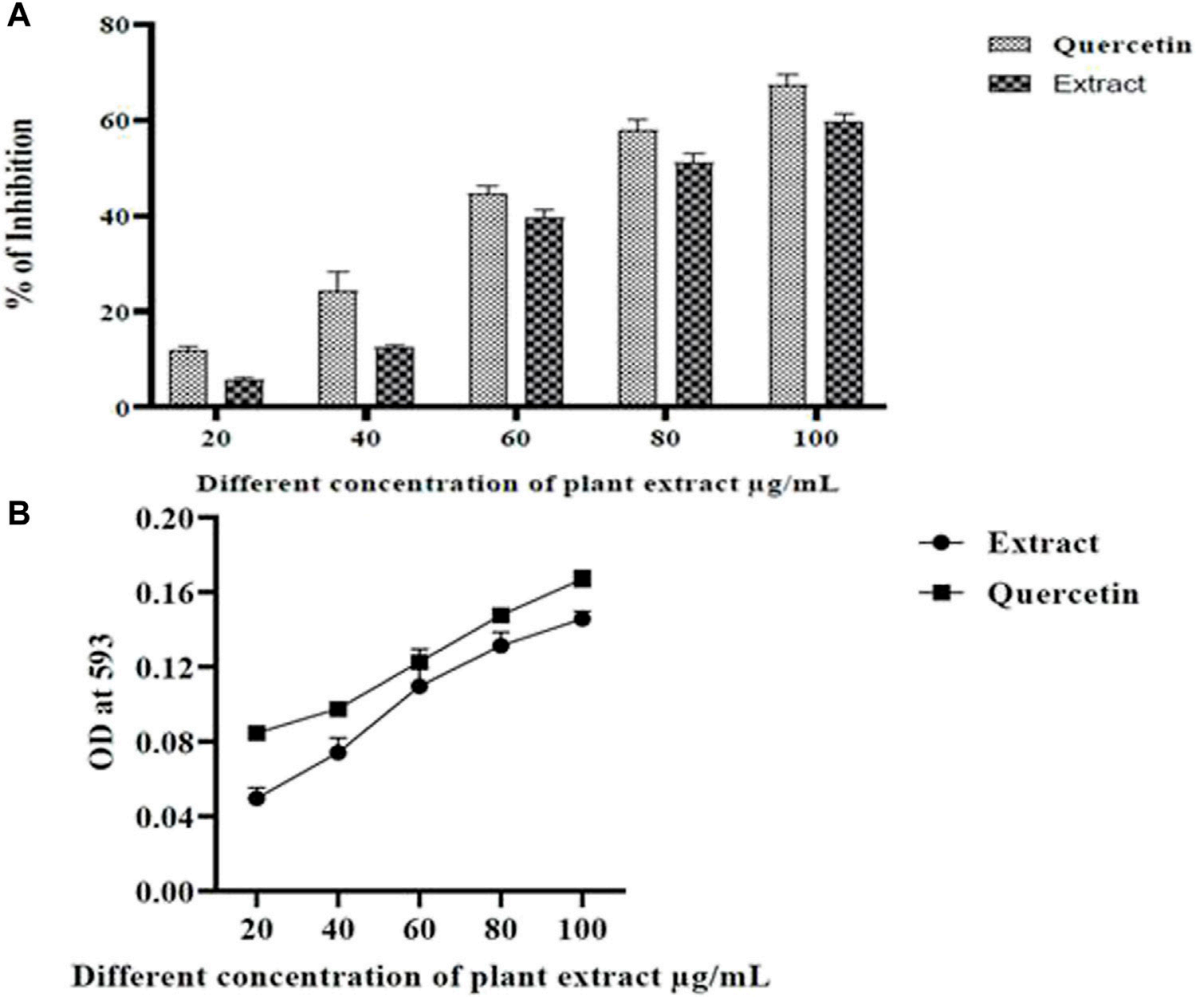
DPPH radical scavenging activity **(A)** and FRAP assay **(B)**.

The oxidative stress markers have induced neurodegeneration in the brain and caused cell damage and cell death (Zhu et al., 2004; Head, 2009). The DPPH assay is stable with free radical absorption at 515 nm when using a colorimetric method while the FRAP assay is stable with free radical absorption at 593 nm when using a spectrophotometric approach. There is a distinct color shift from deep violet to pale yellow. A significant antioxidant activity of the plant extract was also evidenced by the decreased absorbance of the reaction mixture. Good scavenging activity was exhibited by the extract (Figure 3). The most significant scavenging activity was seen in *Olea dioica* Roxb., plant leaf extracts at 100 μg/mL comparable to that of quercetin (Figure 3). As the quantity of the Fe2+-TPTZ complex increased, as observed in the reference antioxidant, the absorbance of leaf extracts increased (Figure 3). Furthermore, the leaf extracts could well be able to donate electrons to free radicals.

### Bioautography assay

**FIGURE 4:**
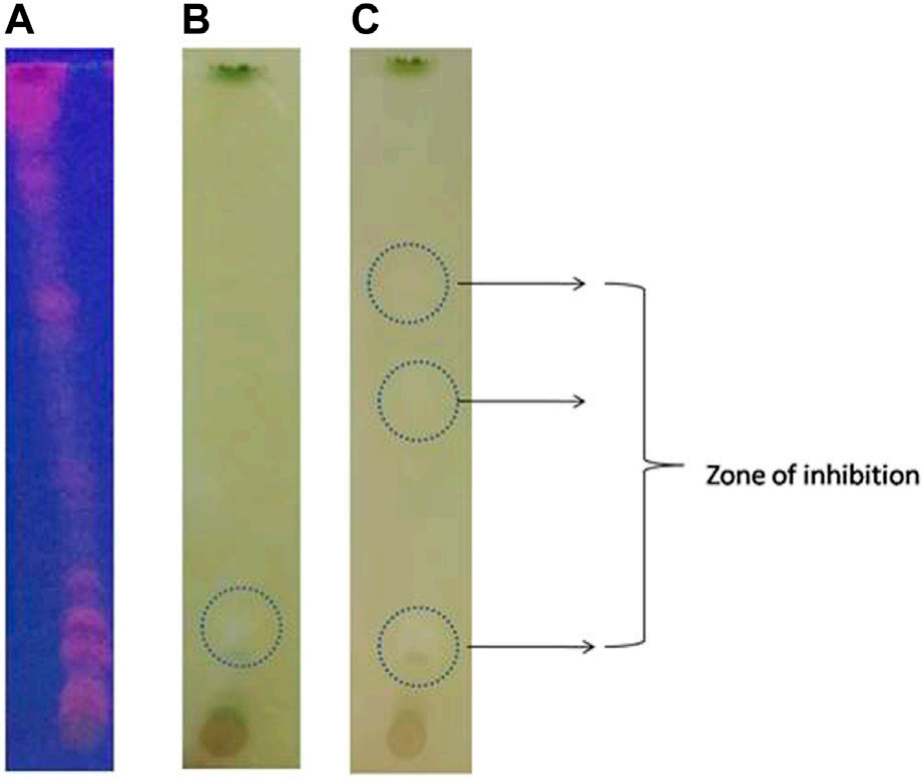
Bio-autographic assay of AChE inhibition activity in the TLC plates. **(A)** TLC plates at UV-light. **(B)** and **(C)** White spots over a yellow background develops as an indication of acetylcholinesterase inhibition zones at different time interval.

Bioautographic enzyme assay is a simple and rapid screening of AChE inhibition by plant extracts (Marston et al., 2002; Pratap et al., 2020a; Pratap et al., 2020b). Our results with methanolic extract of *Olea dioica* showed AChE inhibition on the TLC plate displayingwhite spots or colourless regions of acetylcholinesterase inhibition over yellow background **(** Figure 4 **)**.

### AChE inhibition and ThT binding potential

**FIGURE 5:**
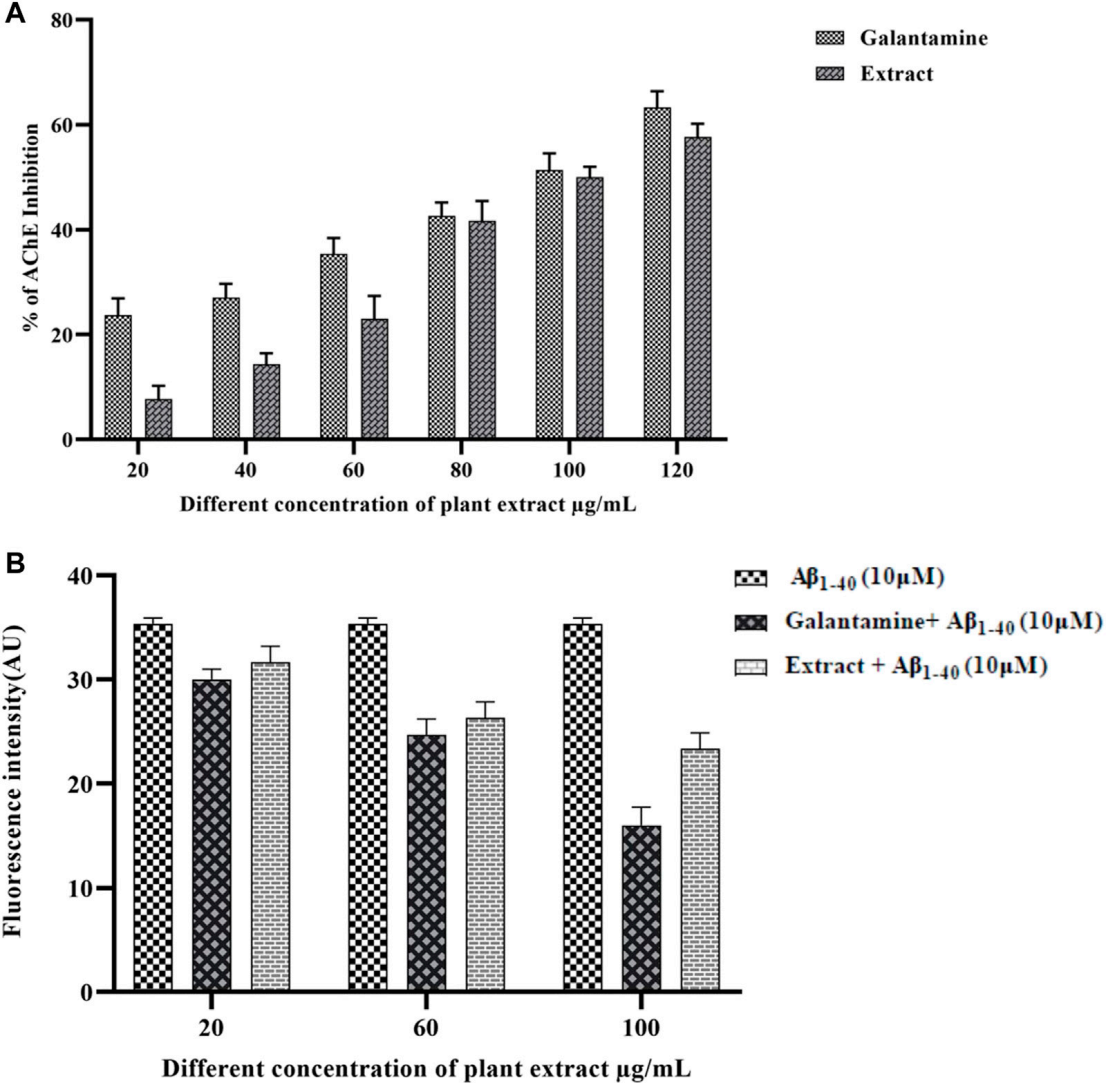
AChE inhibition by plant extracts positive control **(A)** and Thioflavin-T assay of plant extract and positive control **(B)**.

AChE enzyme inhibitory potential of the methanolic extract of *Olea dioica* Roxb. Is shown in Figure 5. The extracts demonstrated strong AChE inhibition, with values > 60% at various sample concentrations (10, 20, 40, 60, 80, and 100 μg/mL). The phenolic compounds are known to be neuroprotective exhibiting AChE inhibitory activity and could be one of the main approaches for the treatment of AD (Pratap and Shantaram, 2020b) (Pratap et al., 2021). The neuroprotective action of the methanolic extract of *Olea dioica* Roxb. Leaves were further assessed by ThT fluorescence assay (Figure 5)**.** ThT binding to amyloid indicated that the methanolic extracts of *Olea dioica* Roxb. Leaves were capable of preventing amyloid-beta aggregation.

### MTT assay and cell viability

**FIGURE 6:**
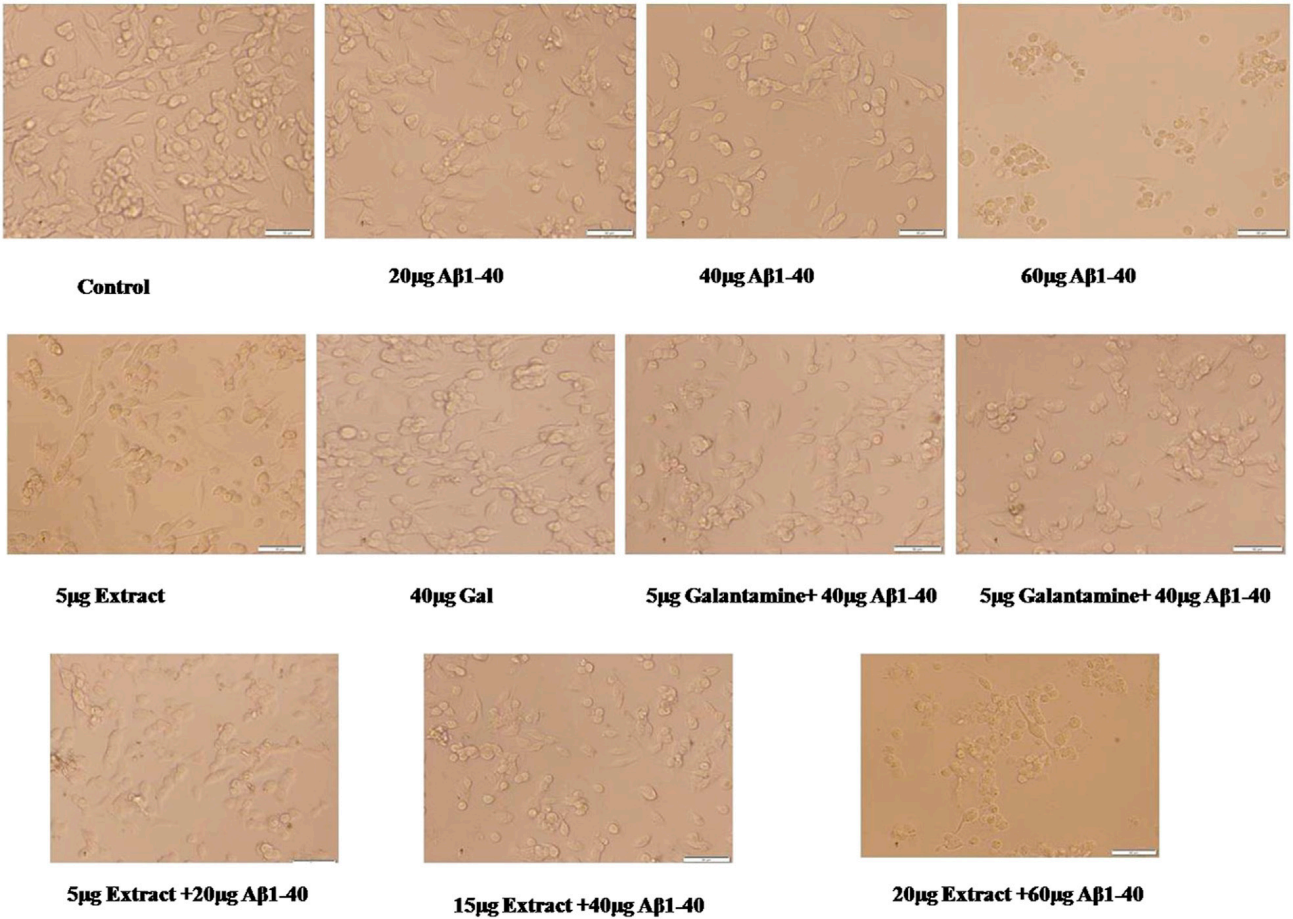
Neuroprotective effect of plant extract against Aβ_1-40_ at different concentrations of inducing cell toxicity in SHSY-5Y cells. Gal (Galantamine) was used as a positive control.

Treatment of Aβ_1-40_ alone at different concentrations (5, 10, and 20 µM) showed different level of cytotoxicity. Aβ_1-40_ concentration (5 and 10 µM) shows apparent cytotoxicity, but Aβ_1-40_ (60 μg/mL) shows a significant cytotoxicity effect on the SHSY-5Y cells. At 10 µMAβ_1-40_concentrations, changes in the cell morphology, cell loss, and cell shrinkage were observed and these changes were analyzed by microscopic examination (Figure 6).

**FIGURE 7:**
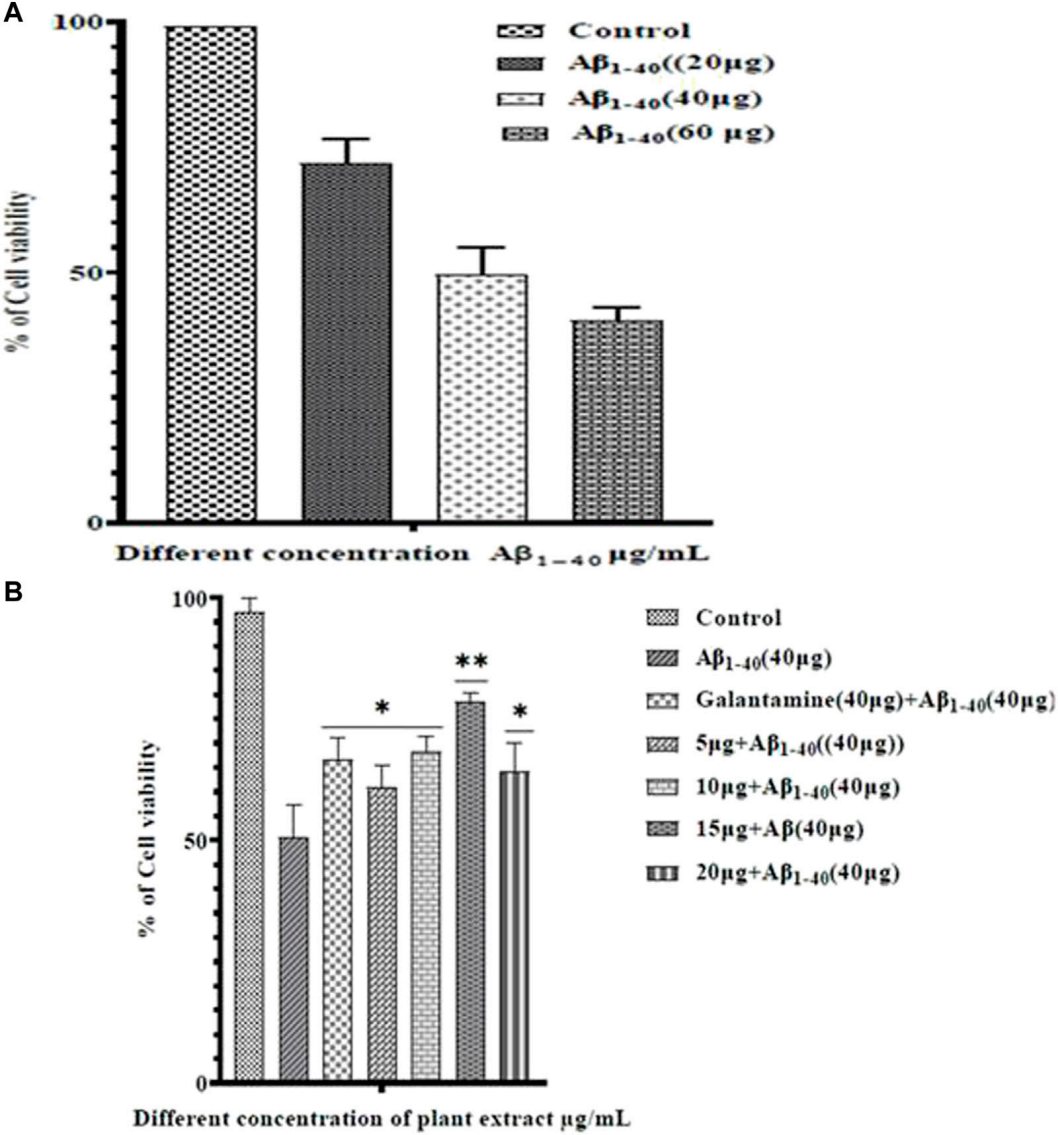
Cell viability in SHSY-5Y cells which were exposed to different concentrations of plant extract for 24 h. Cell viability was assessed by MTT assay.

SHSY-5Y cells were exposed to Aβ_1-40_ (10 µM) with different concentrations of plant extracts (5–20 μM/mL) for 24 h and the cell survival was assessed by MTT assay (Figure 7)**.** Amyloid beta-induced groups have significantly decreased cell survivability with increasing toxicity in a dose-dependent manner. Different concentrations of plant extract along with Aβ_1-40_ (10 µM)treatment groups for 24 h incubation have significantly increased cell survivability compared to the treatment with Aβ_1-40_ (10 µM) alone and confirmed the neuroprotective role.

### ROS activity

**FIGURE 8:**
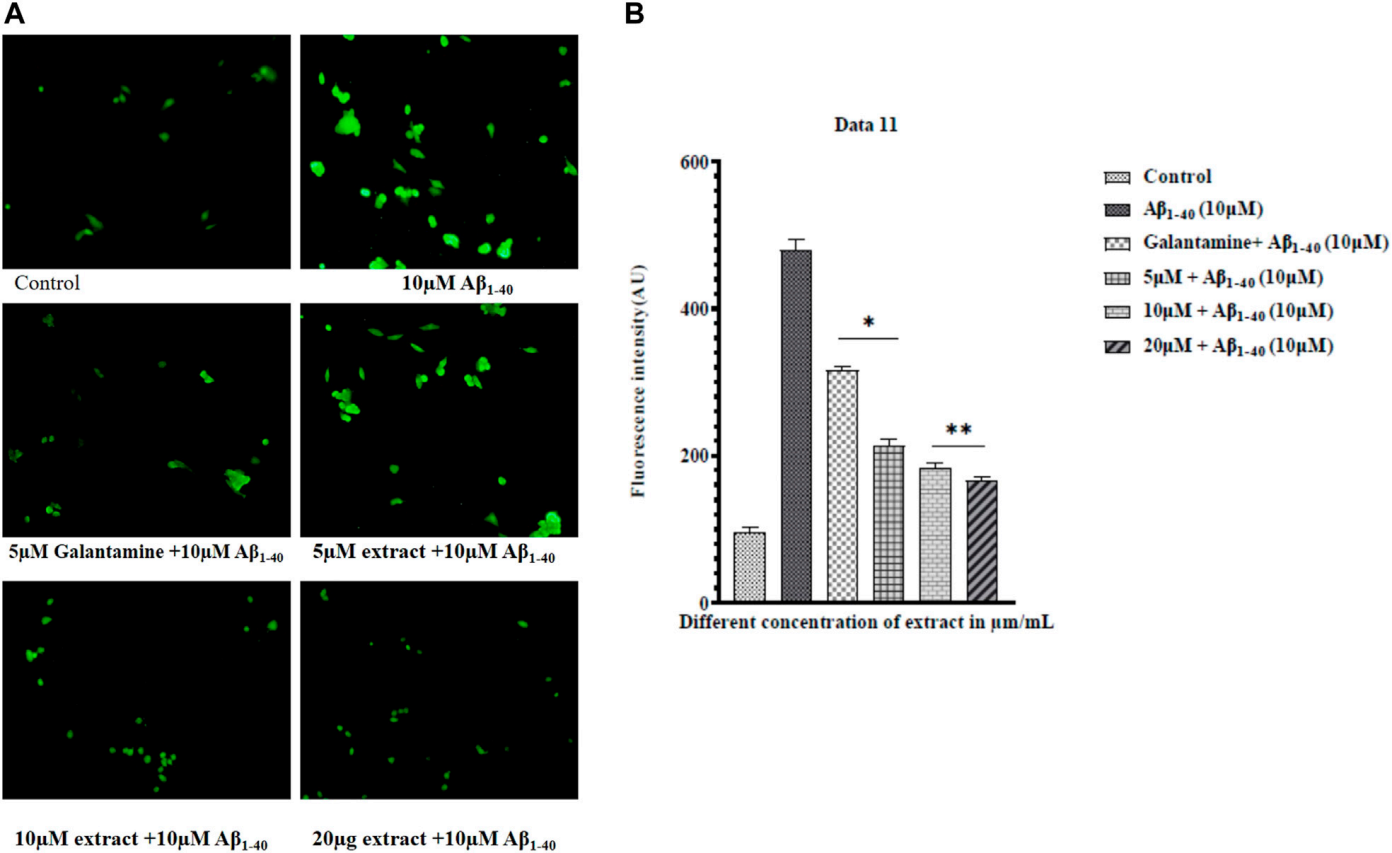
ROS measurement by fluorescence microscope **(A)** and ROS levels in control (Aβ_1-40_) and treated groups of extract + amyloid beta **(B)**. The treated groups decrease the level of ROS in extract treated groups compared to control. The results were expressed in fluorescence intensity. Significance was checked by one-way ANOVA. The error bars represent the Mean ± SD. The *p*-value *p < 0.01 and **p < 0.001, were considered as significant while comparing Aβ_1-40_ treated group and Aβ_1-40_+ extract.

Literature surveys were showcasing that amyloid-beta (Aβ) induced oxidative stress plays a significant role in the progression of AD (Butterfield et al., 2013). Oxidative stress stimulates amyloid-beta production (Adam Moser, Kevin Range and Manuscript, 2008) (H. Wang et al., 2009). The reaction of intracellular ROS with DCFH-DA through fluorescence noticed ROS production. SHSY-5Y cells were exposed to Aβ_1-40_ (10 µM) with extracts and without extracts for 24h, and the ROS level significantly decreased in the Aβ_1-40_ (10 µM) + extract (15 and 20 μM/mL) treatment (Figure 8)**.** The extract at different concentrations significantly prevented Aβ_1-40_ induced ROS production, so the treated group showed a remarkable decrease in the ROS level (Figure 8)**.** Using flow cytometry, the results were obtained. As cellular ROS generation increased, the intensity of the DCFH-DA fluorescence also increased. The extracts at different concentrations inhibited Aβ_1-40_ (10 µM) induced cell toxicity, observed by the shifting of fluorescent intensity, showing the implication of plant extract in preventing the production of ROS.

### Lipid peroxidation assay (LPO)

**FIGURE 9:**
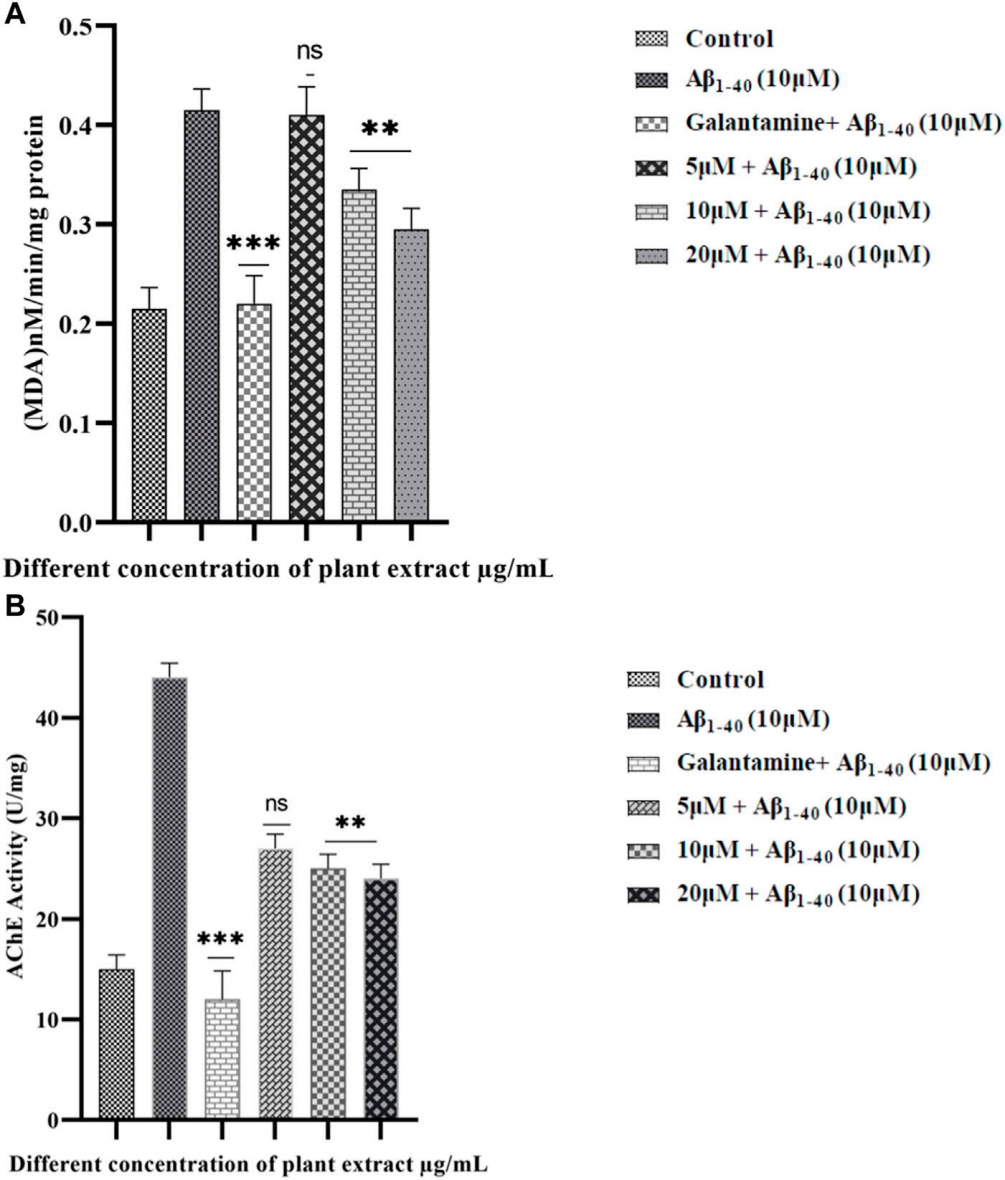
LPO in SHSY-5Y cells were treated with Aβ_1-40_ at different concentrations (5 and 10 µM) **(A).** The effect of extract on treated groups showed a decreasing LPO activity in Aβ_1-40_+ extract treated group. Quantitative evaluation of the extent of inhibition of acetylcholinesterase activity (AChE) by plant extract (5, 15, and 20 μM/mL) and the standard AChE inhibitor galantamine (5 μM/mL) **(B).** The data shown are with Mean ± SD. The *p*-value **p < 0.001, and ***p < 0.001 were considered as significant while comparing Aβ_1-40_ treated group and Aβ_1-40_+ extract.

LPO assay, a distinct indicator of cell death, further supported the protective role of extract. Moreover, the extract could effectively prevent cell injury which can be caused by the Aβ_1-40_ (Figure 9)**.** These study results showed that the extract considerably protected SHSY-5Y cells from the cytotoxicity caused by Aβ_1-40_. Moreover, the LPO level was significantly reduced in the treated group compared to the control group.

### Acetylcholinesterase activity

AChE has played an important role in the implication of the aging processes (Haddadi et al., 2014), where AChE enhances the formation of amyloid-β fibrils and forms the AChE-Aβ complexes which are very neurotoxic (Figure 9). The AChE activity level has increased in SHSY-5Y cells when exposed to Aβ_1-40_ (10 µM) for 24 h. However, the AChE activity level significantly decreased when exposed to Aβ_1-40_ (10 µM) along with the extract (5, 15, and 20 μM/mL) for 24 h.

## Discussion

Bioactive substances found in medicinal plants included phenolics, anthocyanins, and flavonoids. According to reports, the phenolic and flavonoid compounds possess a variety of pharmacological properties, such as the ability to fight cancer, anti-diabetes, and scavenge free radicals. Antioxidants are extremely significant compounds that have the power to shield the body from harm brought on by oxidative stress brought on by free radicals. The phenol and flavonoid contents discovered in leafsample accord with the DPPH results obtained. By virtue of their hydroxyl groups’ ability to donate hydrogen, plant phenols and flavonoids have reducing and antioxidant properties.

The oxidative stress markers cause Parkinson’s disease, and Alzheimer’s disease, as well as neurodegeneration in the brain. According to earlier research on the chemical makeup of various natural compounds, curcumin, Gallic acid, Diterpene, Apigenin, Luteolin, and others are essential for cell division, anti-inflammatory activity, neuroprotection, and anti-amyloidogenic activity. They also act as pro-oxidants (Forni et al., 2019) (Pratap et al., 2021; Gk et al., 2022). According to Haddadi et al. (2014), AChE contributes significantly to the aging process by promoting the production of highly neurotoxic amyloid-b fibrils and AChE-Aβ complexes.

It has been suggested that AChE-Aβ complexes cause more neurodegenerationin the brain than does amyloid-β peptide. AChE is essential for the development of AD (Dhanasekaran et al., 2015). In recent years, the anti-AChE activity of a variety of plant extracts and chemicals produced from plants (Indole, Isoquinoline, Quinolizidine, andPiperidine) has been examined (Devi and Syad, 2014). AChE activity is a widespread biochemical indicator of AD. The current study’s findings revealed that the methanol extract of *O. dioica* contained sizable levels of phenolics and flavonoids. In some medicinal plants such as *Curculigo orchioides* (Pratap et al., 2020a) *Tanacetum parthenium, Nigella sativa* (Chen et al., 2021) *Ginkgo biloba, Bacopa monniera L.)* (Pratap et al., 2017), methanolic extract shows good antioxidant, anti-AChE activity, neuroprotection against amyloid beta toxicity The understudied herb may have a substantial impact on the treatment of human disorders because this leaf extract showed considerable anti-AChE, anti-oxidant, anti-Aβ, and neuroprotective effects.

## Conclusion

As a result of their antioxidant, anti-AChE, and anti-inflammatory qualities, numerous herbal medicines, extracts, phytochemicals, and herbal formulations are said to have anti-Alzheimer’s effects. This results in the decrease of the amyloid beta-induced toxicity. Here our study reports antioxidant, AChE inihibitor, and anti-amyloidogenic activities from methanolic extract of *Olea dioica* Roxb. Leaves aids in neuroprotection. Further detailed investigation of bioactive principles from *Olea dioica* Roxb is needed to establish medicinal values in the control of AD. Based on these results, further studies on the identified bioactive compounds from these two plants are being planned.

## Data Availability

The original contributions presented in the study are included in the article/Supplementary Materials, further inquiries can be directed to the corresponding authors.
